# Immunological parameters of recurrent miscarriages among women in Thi-Qar province

**DOI:** 10.25122/jml-2021-0388

**Published:** 2022-05

**Authors:** Ghaneemah Malik Hamadi, Sally Fadhel Lafta

**Affiliations:** 1. Department of Community Health, Nasiriyah Technical Institute, Southern Technical University, Thi-Qar, Iraq; 2. Public Health Laboratory, Thi-Qar, Iraq

**Keywords:** recurrent miscarriage, autoantibodies, antiphospholipid, cytokines

## Abstract

Recurrent miscarriage (RM) is defined as the loss of pregnancy three or more consecutive times in the first and second trimester, which in some cases occurs due to immune abnormalities. This study aimed to assess some immunological parameters in women with recurrent miscarriages, including the level of antiphospholipid antibody (APA), anticardiolipin (ACA), antinuclear antibody (ANA), complement C3 and C4, and interleukine-3 (IL-3). We included 100 patients together with 100 healthy women as a control. ELIZA was used to measure some types of autoantibodies. APA and ACA significantly increased (P≤0.05) in patients compared to control. In addition, 29% of the patients were positive for antinuclear antibodies (ANA), while the control subjects had negative results for these autoantibodies. Regarding the complement, the serum levels of C3 and C4 were significantly elevated in the serum level of patients when compared to the control group, but in treated patients (heparin and low-dose aspirin), the levels of the complement (C3 and C4) showed a significant decrease in patients compared to total controls. Cytokine level (IL-3) significantly decreased in untreated patients 302.78 pg/ml compared to treated patients (741.57 pg/ml). Antiphospholipid antibodies are more prevalent among women with recurrent miscarriages and are also believed to be the result of abnormal autoimmune activation.

## INTRODUCTION

Recurrent miscarriage (RM) is defined as the loss of two or more consecutive pregnancies affecting approximately 5% of pregnant women worldwide. Recurrent miscarriage is a multifactorial childbearing matter associated with paternal abnormalities in the chromosome, autoimmune disorders, uterine abnormalities, fetal chromosomal rearrangement, endocrine dysfunction, maternal infections, thrombosis, anti-sperm antibodies, and lifestyle factors [1]. However, the exact cause has not been resolved in the remaining 50% of cases [2–3]. Failure of three clinically recognized successive pregnancies before the 20^th^ week of pregnancy is referred to as recurrent miscarriage. However, many reproductive medicine societies define recurrent miscarriage as two or more consecutive pregnancy losses recognized by ultrasound or histopathology [4–5]. Recurrent miscarriage is a common reproductive problem worldwide that concerns 2–5% of pregnant women [6]. It is believed that recurrent miscarriage is a disorder mediated by the immune system [7]. Understanding immune modulation mechanisms during normal pregnancy and recurrent miscarriage have been significantly enriched by modern knowledge about how immune responses work [8]. Antiphospholipid antibodies (APA) bind to phospholipids with a negative charge, and thrombotic events are linked to these antibodies that can cause pregnancy loss. Women with APA (antiphospholipid antibodies) are at risk of pregnancy loss [9]. Previous *in vitro* studies revealed that APA inhibits vascular endothelial growth factor HEEC and its ability to form vessel-like structures [10]. As shown by a systematic review, APA can directly affect trophoblasts' function, leading to placental and vascular impairment transformation [11]. Recurrent miscarriages may be caused by high antiphospholipid antibodies (3–15%) [12]. Antiphospholipid antibodies (APA) have been shown to stimulate blood clotting, activate endothelial cells, and cause fetal loss.

Although the pathogenesis of hepatitis C thrombosis is unknown, it is thought to entail platelets and endothelial cells activation, besides APL's prothrombotic effects on coagulation pathway components [13]. Some studies found a significant association between recurrent spontaneous abortion and the presence of ACA [14]. Another study found that autoantibodies such as antiphospholipid (APA), anticardiolipin antibodies (ACA), and antinuclear antibodies (ANA) play an important role in recurrent pregnancy loss, which is supported by VDRL (the venereal disease research laboratory), prothrombin time (PT) and partial thromboplastin time (PTT) as indirect indicators to find a defect in the immune system of a pregnant woman [15]. APA and ACA positivity among women with recurrent spontaneous abortion are common [16]. Moreover, APA is a significant cause of recurrent trimester miscarriage in women [17]. Another study also found elevated levels of serum anticardiolipin in women with recurrent miscarriages associated with asymptomatic bacterial urine [18]. Some antibodies protect us, while others harm our bodies. The complement system is a component of the innate immune system and one of the effector arms of antibody-mediated immunity [19]. Unregulated activation of complement causes the death of the fetus in mice treated with APL, and APL-mediated coagulation needs complement to increase leukocyte adhesion to the endothelium [20]. Cytokines are actively involved in hematopoiesis by regulating the proliferation, differentiation, and cellular functions of different lineages of hematopoietic cells. Furthermore, cytokines play various roles in normal and abnormal reproduction and pregnancy processes [21]. Interleukins involved in aberrant immunological reactions are particularly noticeable amid countless failures in embryo implantation or events relating to miscarriage. The main cytokine involved in dendritic cell generation is interleukin-3 (IL-3); IL-3 is a hematopoietic growth factor, and it helps in the implantation of the fetus and the generation of the placenta, as the level of the mother's serum rises as a function in trimester [22]. T cells, mast cells, and eosinophils all produce IL-3. Hematopoietic progenitors, neutrophils, macrophages, mast cells, eosinophils, lymphocytes, erythropoiesis, and other cells are all targets [23]. Viaggi et al. [24] suggested that the possible pathogenicity mechanism of antiphospholipid antibodies is known as the capacity of these autoantibodies to suppress the activity of the plasminogen activator. It is reasonable to hypothesize that through its ability to stimulate enzyme activity, IL-3 could support the oval invasion and subsequent implantation, thus preventing early embryo resorption. It was demonstrated that IL-3 plays a role in regulating placenta development. The addition of IL-3 stimulated the growth of placental cells that were not separated *in vitro* in mice. A possible correlation among some immunological markers will therefore be examined among recurrent miscarriage patients in Thi-Qar province. The aim of this study was to assses some immunological parameters in women with recurrent miscarriage.

## MATERIAL AND METHODS

### Study subject

The samples included 100 patients who had recurrent spontaneous abortions positive for primary antiphospholipid antibodies, attending the Bint-AlHuda hospital from February 2021 to July 2021, together with 100 healthy women of reproductive age who never had a miscarriage and without any known immune or rheumatic illnesses as a control. The doctor made the diagnosis, which was determined through clinical assessment and review of the patient's medical history.

### Methods

Phospholipid, Screen IgG/IgM is an ELISA (Enzyme, Immune metric assay) test for detecting IgG and IgM autoantibodies against cardiolipin, phosphatidylserine, phosphatidylinositol, phosphatidyl acid and beta-2-glycoprotein 1 in human serum or plasma. The Demeditec (Germany) ELIZA kit was used. The ANA screen is an ELISA-depend assay technology to qualitatively measure IgG class antibodies against SS-A 60, SS-A 52, SS-B, RNP-70, Sm, RNP/Sm, Scl-70, and centromere B in human serum or plasma, using Demeditec (Germany) kit. The results were calculated according to the protocol of Demeditec (Germany) kits through an index value. An index value less than 1.0 was regarded as negative, while an index value larger than 1.2 was deemed positive. The detection of C3 and C4 protein by radial immunofluorescence plate was performed using Easy RID (Italy). The envelope from the plate was removed and then set aside for a few minutes until any water condensed in the wells had evaporated. 5 µl of sample and/or control liquid was poured into each well. The plate was then sealed with a lid after the sample was spread in a gel for a few minutes, left to stand, and then inverted in the envelope at room temperature for 48 hours. Precipitating ring diameters were measured and compared to the standard table for each immunoglobulin. Serum levels of interleukin-3 (IL3) were assessed in patients and control groups using ELISA kits for the quantitative determination of cytokines (IL-3) in serum (Ray Bio, USA).

### Statistical Analysis

The statistical analysis was carried out using SPSS version 23. Data are presented as mean and standard error (SE). A comparative analysis of the patients and control was conducted using a T-test. The results were considered statistically significant at a level of P≤0.05.

## RESULTS

This research included patients with recurrent miscarriage (100) and 100 controls. After the initial age stratification, 30% were in the 20–25 years category, 38% were 26–30, 19% were 31–35, and 13% were 36–40. APA IgG/IgM was elevated in the serum of patients with recurrent miscarriages (11.13±1.12, 21.14±1.22 IU/ml) compared with controls (1.49±0.12, 1.89±0.13 IU/ml) (P≤0.05) (Table 1).

**Table 1:** Antiphospholipid antibody (APA) level in serum of patients and controls.

APA(IU/ml)	Mean±SE		P-value
	Patients	Controls	
**IgG**	1.12±11.13	1.49±0.12	<0.05
**IgM**	1.22±21.14	1.89±0.13	

ACAIgG/IgM was elevated in the serum of patients (9.22±2.15, 20.31±1.82 IU/ml) compared with controls (1.51±0.26, 1.41±0.12 IU/ml) (P≤0.05) (Table 2).

**Table 2:** Anticardiolipin antibody (ACA) level in the serum of patients and controls.

APA(IU/ml)	Mean±SE		P-value
	Patients	Controls	
**IgG**	2.15±9.22	1.51±0.26	<0.05
**IgM**	1.82±20.31	1.41±0.12	

The serum of patients with recurrent miscarriage and controls was evaluated for autoantibodies using the ANA assay. 29% of the total patients were positive for ANA, while the serum of the control subjects was negative for these autoantibodies (Table 3).

**Table 3:** Antinuclear antibody (ANA) level in the serum of patients and controls.

Groups	Patients		Controls	
	No	%	No.	%
**ANA positive**	27	27.0	0	0.00
**ANA negative**	73	73.0	100	100
**Total**	100		100	

Our results illustrated that complement C3 had a non-significant decrease in patients with treatment (low dose aspirin and heparin) compared to the control group (89.57 *vs*. 96.17), and in C4 there was a significant decrease in patients compared to the control group (11.89 *vs*. 29.38). In contrast, a significant increase in C3 and C4 levels was observed in patients without treatment compared to the control group (111.19 *vs*. 96.17), (34.41 *vs*. 29.38), respectively (Table 4).

**Table 4:** Concentration Complements C3 and C4 in serum of patients and controls.

Groups	Patients with treatments	Patients without treatments	Controls
**Level of C3 (mg/dl) Mean±SD**	6.21±89.57	10.24±111.19	2.68±96.17
**Level of C4 (mg/dl) Mean±SD**	1.22±11.89	2.52±34.41	0.73±29.38

Our results showed a decreased level of IL-3 in patients without treatment (302.78pg/ml) compared to controls (624.12 pg/ml), also compared to patients treated with heparin and low-dose aspirin (741.57 pg/ml) (Table 5).

**Table 5:** Concentration of IL-3 in patients and controls.

Groups	Patients with treatments	Patients without treatments	Controls
**level of IL-3 (pg/ml) Mean±SE**	48.05±741.57	27.18±302.78	59.36±624.12

## DISCUSSION

Spontaneous abortion is the most frequent pregnancy problem, occurring in 10–15% of pregnancies in the first trimester [25]. The mechanisms of pregnancy loss are not totally understood. Immunological rejection of the fetus by the mother's immune system is thought to be involved, in addition to chromosomal, embryonic, and morphological abnormalities. The current study showed that APA IgG/IgM was elevated in the serum of patients with recurrent miscarriage compared with controls and the percentages of positivity for APL (IgM) were higher than for APL (IgG). This was confirmed by Ilyas et al. [26] who found that the percentages of positivity for APL (IgM) are higher than for APL (IgG). Recurrent pregnancy failure was also associated with an increased prevalence of several autoantibodies, including antiphospholipid antibodies (APA). Our findings revealed that the level of anticardiolipin antibodies (IgM, IgG) in patients was significantly higher than the corresponding value in the control group.

This was in agreement with Jawad et al. [12] and Al-Jabery [15] that anticardiolipin autoantibodies are responsible for recurrent pregnancy loss, fetal growth restriction, uterine insufficiency, infarction, arrhythmias, and, finally, stillbirth. The antinuclear antibody (ANA) in this study was positive (29%). This was consistent with the finding of Al-Jaberi [15], who found that 20.4% was ANA positive. Antinuclear antibodies cause immune problems in 22% of women with multiple pregnancies and 50% of infertile women who had IVF failures. In the fetus or during pregnancy, women who have this issue produce DNA antibodies or breakage products. These antibodies start in the form of IgM in the bloodstream. As the condition worsens, they manifest as IgG and are found in the lymphatic system and lymph nodes.

With additional losses, they form IgA antibodies, which are present and function in organs such as the uterus. These antibodies can be directed against clear double-stranded DNA (dsDNA), single-stranded DNA (ss DNA), or smaller molecules known as polynucleotides, and histones comprise the single strands [27]. Apart from the antinuclear antibody (ANA), the antiphospholipid antibody is the most prevalent antibody associated with myelitis, being positive in 43 to 73% of myelitis patients. On the other hand, antiphospholipid antibodies are not over-represented in patients with SLE who have myositis. Consequently, it is uncertain whether antiphospholipid antibodies have a pathogenic role in SLE, while some writers have postulated that antiphospholipid antibodies can cause a venous infarction, which can lead to SLE thrombotic myelopathy [28].

In patients with antiphospholipid antibodies, antinuclear antibodies have a positive effect on antinuclear antibody (ANA), mottled pattern, and DNA autoantibodies lead to inflammation in the placenta, and the autoimmune disease examination in women is negative. Women suffering from lupus, Crohn's disease, rheumatoid arthritis, and other autoimmune disorders have positive antibodies. In this study, the third (C3) and fourth (C4) complement levels showed an increase in patients with RM compared to controls. This result is consistent with the study by Sikara et al. [29] who found the same results. APL induces an inflammatory and prothrombotic phenotype in endothelial cells, monocytes, and platelets. It is also well documented that the complementary activating components can bind to and activate inflammatory cells and endothelial cells, either directly through the C5b-9 transmembrane attack complex (MAC) or through C5a receptor-mediated effects.

Furthermore, endothelial cells release TF upon activation by Anaphylatoxins C5a [29]. Chaturvedi et al. [30] found that patients with RM tended toward increased complement activation. In the current study, during the treatment of patients, the level of complement III (C3) and IV (C4) showed a decreased antiphospholipid antibody compared to controls.

This result is in agreement with the study of Chiara et al. [31] who found that heparin and dose aspirin are nowadays the gold standard for preventing obstetric complications in pregnant women with APA by increasing trophoblastic cell invasion and differentiation and endometrial angiogenesis. At the same time, there is a decrease in both anti-APA binding with trophoblast cells, complement activation, and apoptosis of trophoblast cells.

Samarkos et al. [32], showed that antibodies against APL specifically target the placenta and induce activation of platelets and endothelial cells, leading to the prothrombotic condition. However, this is not enough to cause fetal loss or growth retardation. Activation of complement increases the production of mediators such as C3a, C5a, and C5b-9 MAC, which promote activation of platelets and endothelial cells, leading to inflammation, tissue damage, and finally, fetal loss.

Interleukin-3 is the main cytokine in dendritic cell formation and plays an important role in dendritic cell formation. Interleukin-3 stimulates (Th2) T helper 2 cell responses, DC alterations of monocyte-derived cell responses to the Th2 cytokine pattern. Thus, IL-3 activity may affect the homeostasis of Th1 and Th2 cytokines. A cytokine level imbalance in Th1/Th2 cells is a significant risk factor for the miscarriage of the fetus. Recurrent miscarriage is associated with Th1, while healthy pregnancy is associated with Th2 [33].

A significantly high level of the Th1/Th2 cytokine is present in infertile women with multiple implantation failures after IVF and women with recurrent pregnancy loss [14]. IL-13 is a growth factor for trophoblasts that assists embryo implantation and placental development [12]. The results of the current study showed that IL-3 was reduced in RM patients compared to controls. IL-3 may be effective in preventing recurrent fetal loss.

These results are in agreement with the study of Wu. et al. [34] who found that IL-3 was associated with the risk of miscarriage. The current study is also in agreement with Schwat et al. [35] who found that treatment with IL-3 prevents miscarriage. Fishman et al. [36] also showed that APL mice are also IL-3-deficient and surprisingly responsive to exogenous IL-3, and a possible mechanism is that IL-3 stimulates trophoblast growth sufficiently to compensate for placental ischemic atrophy. Relatively, thrombocytopenia and lupus anticoagulants returned to normal in the treated animals. The mechanism by which IL-3 reverses the effects of APL on pregnancy is not fully understood. Tersigni et al. [31] presented that IL-3 can reverse the effects of APL on hormone secretion and trophoblast invasion. However, Chamley and others question if it has the same effects on human trophoblasts [37].

## CONCLUSION

Antiphospholipid antibodies are more prevalent among women with recurrent miscarriages and are also believed to be the result of abnormal autoimmune activation. Significant elevation of C3 and C4 serum levels were found in patients. These results showed that tissue injury in the MR is due to a complement-mediated inflammatory process. In this study, a decreased level of IL-3 was detected in RM patients without treatment, and an increase in the patients with treatment provided that IL-3 was effective in preventing recurrent fetal loss.
